# Noonan Syndrome in South Africa: Clinical and Molecular Profiles

**DOI:** 10.3389/fgene.2019.00333

**Published:** 2019-04-16

**Authors:** Cedrik Tekendo-Ngongang, Gloudi Agenbag, Christian Domilongo Bope, Alina Izabela Esterhuizen, Ambroise Wonkam

**Affiliations:** ^1^Division of Human Genetics, Departments of Medicine and Pathology, Faculty of Health Sciences, University of Cape Town, Cape Town, South Africa; ^2^Departments of Mathematics and Computer Sciences, Faculty of Sciences, University of Kinshasa, Kinshasa, Democratic Republic of Congo; ^3^National Health Laboratory Service, Groote Schuur Hospital, Cape Town, South Africa; ^4^Faculty of Health Sciences, Institute of Infectious Diseases and Molecular Medicine, University of Cape Town, Cape Town, South Africa

**Keywords:** Noonan syndrome, multigene panel testing, targeted next-generation sequencing, RASopathies, Ras/MAPK signaling pathway, South Africa

## Abstract

Noonan Syndrome (NS) is a common autosomal dominant multisystem disorder, caused by mutations in more than 10 genes in the Ras/MAPK signaling pathway. Differential mutation frequencies are observed across populations. Clinical expressions of NS are highly variable and include short stature, distinctive craniofacial dysmorphism, cardiovascular abnormalities, and developmental delay. Little is known about phenotypic specificities and molecular characteristics of NS in Africa. The present study has investigated patients with NS in Cape Town (South Africa). Clinical features were carefully documented in a total of 26 patients. Targeted Next-Generation Sequencing (NGS) was performed on 16 unrelated probands, using a multigene panel comprising 14 genes: *PTPN11, SOS1, RIT1, A2ML1, BRAF, CBL, HRAS, KRAS, MAP2K1, MAP2K2, NRAS, RAF1, SHOC2*, and *SPRED1*. The median age at diagnosis was 4.5 years (range: 1 month−51 years). Individuals of mixed-race ancestry were most represented (53.8%), followed by black Africans (30.8%). Our cohort revealed a lower frequency of pulmonary valve stenosis (34.6%) and a less severe developmental milestones phenotype. Molecular analysis found variants predicted to be pathogenic in 5 / 16 cases (31.2%). Among these mutations, two were previously reported: *MAP2K1*-c.389A>G (p.Tyr130Cys) and *PTPN11* - c.1510A>G (p.Met504Val); three are novel: *CBL*-c.2520T>G (p.Cys840Trp), *PTPN11*- c.1496C>T (p.Ser499Phe), and *MAP2K1*- c.200A>C (p.Asp67Ala). Molecular dynamic simulations indicated that novel variants identified impact the stability and flexibility of their corresponding proteins. Genotype-phenotype correlations showed that clinical features of NS were more typical in patients with variants in *MAP2K1*. This first application of targeted NGS for the molecular diagnosis of NS in South Africans suggests that, while there is no major phenotypic difference compared to other populations, the distribution of genetic variants in NS in South Africans may be different.

## Introduction

Noonan syndrome (NS; MIM 163950) is a common autosomal dominant condition, with an estimated global incidence of 1:1,000 to 1:2,500 live births (Mendez and Opitz, 1985). Affected individuals present with multisystem involvement, including short stature, distinctive craniofacial dysmorphism, congenital heart defects (CHD), skeletal abnormalities, developmental delay, coagulation defects, and other abnormalities (Allanson and Roberts, 2016). Noonan syndrome is clinically heterogeneous with significant interfamilial and intrafamilial variable expression. Noonan syndrome condition is caused by heterozygous germline mutations in more than 10 genes encoding, either proteins of the Ras family of GTPases (*KRAS, NRAS, RIT1*, and *RRAS*), or modulators of Ras function (*PTPN11, SOS1, SOS2, CBL, RASA2*, and *SHOC2*) and downstream signal transducers (*RAF1, BRAF*, and *MAP2K1*) (Cordeddu et al., 2015; Aoki et al., 2016). To date, mutations in all identified genes for NS result in gain-of-function within the Ras/MAPK pathway, and account for up to 80% of NS cases (Aoki et al., 2016). In many clinical settings, missense variants in *PTPN11* alone are found in about 50% of affected individuals (Tartaglia and Gelb, 2005), while *SOS1* has been reported to be the second most mutated gene, accounting for 10–20% of *PTPN11*-negative patients (Roberts et al., 2007). Compared to *PTPN11* and *SOS1*, the contribution of other known genes seems to be minimal, with variable mutation frequencies observed across populations (Allanson and Roberts, 2016). Recent studies have reported association between biallelic variants in *LZTR1* and NS phenotype (Johnston et al., 2018; Nakaguma et al., 2019), supporting the existence of an autosomal recessive form of the condition, as suggested by some clinical studies (Abdel-Salam and Temtamy, 1969; Maximilian et al., 1992; Van Der Burgt and Brunner, 2000). However, heterozygous variants in *LZTR1* have also been previously associated to the NS phenotype in at least seven families (Chen et al., 2014; Yamamoto et al., 2015), and the consequences of *LZTR1* pathogenic variants on the Ras/MAPK signaling pathway remain to be clarified. The clinical diagnosis of NS is often straightforward in several cases, through recognition of key craniofacial and musculoskeletal features, in combination with CHD (Romano et al., 2010). Phenotypic overlap with other conditions sharing the same pathogenetic mechanisms so-called RASopathies, challenges the diagnosis of NS in some patients and families; this is particularly true for Cardiofaciocutaneous syndrome (CFC) and Costello syndrome. As evidenced by several genetic studies, phenotypic expressivity in many conditions may show variations across populations and ethnic groups, making it more challenging to apply well-established clinical diagnostic criteria universally (Tekendo-Ngongang et al., 2014; Kruszka et al., 2017). Affected individuals with NS from sub-Saharan Africa have seldom been reported in the literature (Kruszka et al., 2017). Furthermore, no study investigating the genetic etiology of NS in South Africans has been previously conducted, despite that numerous individuals and families affected with NS were identified in South Africa, largely due to unavailability of molecular diagnostic testing for RASopathies in the state public sector. The present study aimed at characterizing a cohort of South African patients with NS from clinical and molecular perspectives, using targeted NGS approach.

## Materials and Methods

### Ethical Approval

The study was performed in accordance with the Declaration of Helsinki and with the approval of the Faculty of Health Sciences Human Research Ethics Committee, University of Cape Town (HREC: 449/2016). Written informed consent was obtained from the parents and/or the patient prior to their involvement into the study, including permission to publish photographs.

### Patients and Phenotyping

This study was conducted in Cape Town, South Africa, and included 26 participants (20 children and six adults); among them, 20 were unrelated. Patients were recruited through the University of Cape Town (UCT) affiliated Hospitals, namely Red Cross War Memorial Children's Hospital (RCWMCH) and Groote Schuur Hospital (GSH). Patients were selected retrospectively and prospectively using the Van der Burgt scoring system for clinical diagnosis of NS (Van der Burgt, 2007). All pediatric and adult patients were assessed by trained clinical geneticists familiar with RASopathies. For each proband, family history suggestive of NS was systematically assessed by means of three or more generations pedigree. Phenotypic data recorded included all clinical characteristics, with emphasis on antenatal features, NS-specific dysmorphology assessment, investigation of bleeding diathesis and cardiovascular abnormalities on Electrocardiograms (ECG) and echocardiograms.

### Molecular Methods

Genomic DNA of each selected patient was isolated from peripheral blood leukocytes at the National Health Laboratory Service (NHLS)-Molecular Genetics Laboratory, GSH, following the manufacturer's instructions, the standard Maxwell 16 protocol (Maxwell®16 Blood DNA purification kit, Promega, Madison, WI 53711, USA).

### Targeted Gene Panel Sequencing and Variants Analysis

Of the 26 DNA samples isolated, 16 DNA samples from unrelated patients were genotyped. Targeted Sequencing was performed with the Ion Torrent platform, at the sequencing laboratory of the Division of Human Genetics of UCT, using the Ion PGM™ system (Thermo Fisher Scientific, Waltham, Massachusetts, USA). Pre-designed primers for Ion AmpliSeq Noonan Research Panel were used (Life Technologies, Carlsbad, CA). The primers amplify exons and intron/exon boundaries of 14 genes known to be associated with NS and related conditions, including *A2ML1, BRAF, CBL, HRAS, KRAS, MAP2K1, MAP2K2, NRAS, PTPN11, RAF1, RIT1, SHOC2, SOS1*, and *SPRED1*. This multigene panel is predicted to cover 100% of the targeted regions, in 268 amplicons (Nelen et al., 2014). Sequencing data analysis, including quality assessment, read alignment, variants identification, variant annotation, visualization, and prioritization was primarily performed using the bioinformatics pipeline of the Ion Torrent Suite and the Ion Reporter cloud-based software (Thermo Fisher Scientific, Waltham, Massachusetts, USA). From the usable reads, 99% could be mapped to the human reference genome used (Homo sapiens, hg19, build 37.2). Further manual analysis was executed for variant prioritization and interpretation based on the variant call format (VCF) file generated by the Ion Reporter software. In this step, variants were prioritized using their minor allele frequency (MAF < 0.01) based on 1,000 genomes and 5,000 exomes projects, their zygosity, their function, their location within the gene, and their pathogenicity according to ClinVar. A parallel analysis of sequencing data was performed based on the binary alignment map (BAM) file generated by the Ion Reporter software. Picard package with option to SortSAM, MarkDuplicates and FixMateInformation on a per-sample basis were used to sort coordinate, mark polymerase chain reaction (PCR) duplicate reads and verify mate-pair information, respectively (Mckenna et al., 2010). The variant calling was done using Samtools, bcftools (Li et al., 2009) and Varscan 2 (Koboldt et al., 2012) with the reference Human genome (Hg19; build 37.2). The conservation and deleteriousness of the variant were investigated using ANNOVAR which interrogated the following tools: SIFT, PolyPhen 2 HVAR, Polyphen2 HDIV, MutationTaster, MutationAssessor, Likelihood ratio test (LRT), FATHMM, MetaSVM, MetaLR, GERP++, PhyloP, VEST3, DANN, CADD, PROVEAN, Fathmm-MKL, Integrated_fitCons, SiPhy_29way, PhastCons (Wang et al., 2010). The second level of variant filtration to avoid false positives or false negatives was conducted on annotated VCF files using an in-house python script to select and retain only deleterious disease-causing variants that have functional prediction using the 19 tools interrogated by ANNOVAR. The in-house python script uses two approaches to select deleterious variant (i) free hypothesis: cast of the vote of the annotated variant filter for “Deleterious or damaging disease-causing (D)” among annotation prediction tools based on a defined cut-off (~50%); (ii) non free hypothesis: provide a list of known genes of the study with another level of prediction cut-off (~25%) (Lebeko et al., 2017). The cut-off for both hypothesis is defined as follow; (i) free hypothesis: select only variant which 10 tools interrogated by ANNOVAR predicted presence of a “D”; (ii) no free hypothesis: select only the gene which 5 tools interrogated by ANNOVAR predicted presence of a “D” using the list of 14 NS genes analyzed. Existing online databases for previously reported NS variants and published literature on NS-associated variants were consulted for each candidate variant.

### Sanger Sequencing Validation and Family Segregation Studies

All variants identified and predicted to be pathogenic were subsequently confirmed with Sanger capillary direct cycle sequencing and capillary electrophoresis using standard protocols. Depending on availability of family members, segregation studies were performed: the proband's parents were screened to ascertain the origin of the variant. In addition, and where possible, other available family members clinically affected or not, were screened for the identified variant, using Sanger direct cycle sequencing.

### 3D Protein Structure Prediction for Functional Characterization of Novel Variants

Molecular dynamic (MD) simulations were conducted to assess the effect of novel variants on proteins function. The tertiary structure of the *PTPN11* protein SHP-2 (PDB id: 2SHP) (Hof et al., 1998) and the MAP2K1 structure (UniProKD: A4QPA9) were generated using I-tasser homology webserver (Zhang, 2008). The CBL structure was generated by combining (PDB id: 1FBV) and homology model. Six independent MD simulations were conducted; (i) MAP2K1 mutant and wild-type (WT), SHP-2 mutant and WT, and CBL mutant and WT. All MD simulations were conducted with the GROMACS package, version 4.6.5 (Pronk et al., 2013) using OPLS force field (Jorgensen and Tirado-rives, 1988). The systems were simulated in cubic box and solvated in water TIP3P (Neira et al., 1996). The temperature and pressure were maintained at 300 K using the Parrinello-Donadio-Bussi V-rescale thermostat (Bussi et al., 2007) and a pressure of 1 bar using the Berendsen barostat (Berendsen et al., 1984). The short-range non-bonded interactions were modeled using Lennard Jones potentials. The long-range electrostatic interactions were calculated using the particle mesh Ewald (PME) algorithm (Darden et al., 1993; Essmann et al., 1995). The LINCS algorithm was used to constrain all bond lengths (Hess et al., 1997) and the velocities were assigned according to the Maxwell-Boltzman distribution at 300 K.

## Results

### Socio-Demographics

A total of 26 patients were included in this study, mostly unrelated (*n* = 20; 77%). The majority were children *(n* = 20; 77%) with a median age at clinical diagnosis of 4.5 years (range: 1 month−51 years). Our cohort had preponderance of individuals of mixed-ancestry background (53.8%), followed by black Africans (30.8%). There was a slight predominance of males (sex ratio: 1.3; 15 males: 11 females).

### Phenotypic Description

Gross motor milestones were on par for most patients, with ability to walk before the age of 18 months in 61.5% (*n* = 16/26) of cases. Six (6/26; 23.1%) patients were unable to walk after 24 months. Speech delay was reported in 50% (13/26) of cases. Craniofacial features were widely variable, but more characteristic in infants (2–12 months; 4/26), with widely spaced eyes, epicanthic folds and ptosis found in 75% of cases (Table 1). Comparison of six key physical characteristics (short stature, ptosis, widely spaced eyes, epicanthic folds, low-set ears, and webbed neck) between ethnic groups showed that, features were less frequent in Caucasians (2/26). Black Africans (8/26) presented with the most consistent dysmorphic features, with epicanthic folds (87.7%), ptosis (75%), and low-set ears (75%) found to be the most common features (Table 2). At least one cardiovascular abnormality was identified in 65.4% (17/26) of patients. The most common CHD was pulmonary valve stenosis (PS), found in 34.6% (9/26) of our cohort. Hypertrophic Cardiomyopathy (HCM) was identified in 19.2% (5/26) and left axis deviation on ECG was found in 23.1% (6/26) of cases. The complete list of clinical features identified in our cohort of 26 patients can be found in the Table S1.

**Table 1:** Comparison of craniofacial features between age groups in the cohort of 26 patients.

**Features**	**New-born (*n* = 1)**	**2–12 months (*n* = 4)**	**1–12 years (*n* = 15)**	**>18 years (*n* = 6)**
Macrocephaly	1 (100%)	2 (50%)	0	0
Tall and prominent forehead	1 (100%)	2 (50%)	8 (53.3%)	1 (16.7%)
Coarse face	0	1 (25%)	2 (13.3%)	5 (83.3%)
Elongated face	1 (100%)	1 (25%)	6 (40%)	4 (66.7%)
Widely spaced eyes	0	3 (75%)	5 (33.3)	0
Epicanthic folds	1 (100%)	3 (75%)	9 (60%)	3 (50%)
Ptosis	0	3 (75%)	10 (66.7%)	0
Low-set ears	1 (100%)	2 (50%)	9 (60%)	3 (50%)
Short, broad, depressed nasal root	0	3 (75%)	12 (80%)	3 (50%)
Prominent naso-labial folds	0	0	2 (13.3%)	2 (33.3%)
High wide peaks of the vermilion	0	2 (50%)	11 (73.3)	2 (33.3%)
Short neck	0	2 (50%)	8 (53.3%)	2 (33.3%)
Webbed neck	0	1 (25%)	3 (20%)	1 (16.7%)

**Table 2:** Comparison of six key dysmorphic features between ethnic groups.

**Features**	**Black african (*n* = 8)**	**Mixed-race ancestry (*n* = 14)**	**Caucasian (*n* = 2)**	**Indian (*n* = 2)**
Widely spaced eyes	2 (25%)	5 (35.7%)	0	1 (50%)
Ptosis	6 (75%)	7 (50%)	0	0
Epicanthic folds	7 (87.5%)	9 (64.2%)	0	1 (50%)
Low-set ears	6 (75%)	7 (50%)	1 (50%)	1 (50%)
Webbed neck	3 (37.5%)	1 (7%)	1 (50%)	0
Short stature	7 (87.5%)	12 (87.5%)	0	2 (100%)

### Genetic Variants Profile

**Figure 1:**
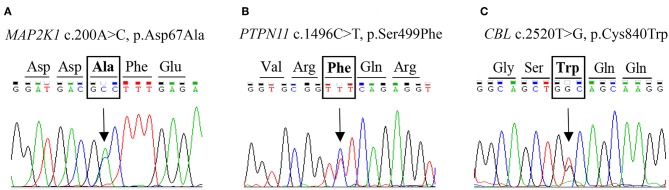
Electropherograms showing three novel heterozygous missense variants. The arrow indicates location of the base pair substitution in: **(A)** *MAP2K1* gene, **(B)** *PTPN11* gene, and **(C)** *CBL* gene.

Following targeted NGS of 16 DNA samples from unrelated patients, *in silico* predictive algorithms supported the classification of seven heterozygous missense variants as pathogenic (7/16; Table 3; Figure 1). The novel variants were *CBL* c.2520T>G (p.Cys840Trp), *PTPN11* c.1496C>T (p.Ser499Phe), and *MAP2K1* c.200A>C (p.Asp67Ala). Analysis confirmed segregation of three novel variants with the disease in the respective families. Segregation analysis revealed a possible case of germline mosaicism in a family where the unaffected father does not carry the variant (*MAP2K1*; c.200A>C) found in his two affected children. These children were half-brothers, born from two separate mothers.

**Table 3:** Characteristics of pathogenic variants identified.

**Locus**	**Transcript**	**Gene**	**Exon**	**Nucleotide substitution**	**Amino acid substitution**	**Variant function**	**Family status**	**ClinVar variation ID**	**Associated disease**
chr15:66729181	NM_002755.3	*MAP2K1*	3	c.389A>G	p.Tyr130Cys	Missense	Unknown	13351	CFC
chr15:66727484	NM_002755.3	*MAP2K1*	2	c.200A>C	p.Asp67Ala	Missense	Familial	Not in ClinVar	NS
chr11:119170290	NM_005188.3	*CBL*	16	c.2520T>G	p.Cys840Trp	Missense	Familial	Not in ClinVar	NS
chr12:112926890	NM_002834.3	*PTPN11*	13	c.1510A>G	p.Met504Val	Missense	Unknown	40562	NS
chr12:112926876	NM_002834.3	*PTPN11*	13	c.1496C>T	p.Ser499Phe	Missense	Familial	Not in ClinVar	NS

The two variants previously reported in ClinVar as pathogenic are *MAP2K1* c.389A>G (p.Tyr130Cys) and *PTPN11* c.1510A>G (p.Met504Val). Two identified *CBL* variants were predicted to be pathogenic by prediction tools, but were reported as benign: c.1858C>T (p. p.Leu620Phe) and as variant of uncertain significance: c.2345C>T (p.Pro782Leu) in ClinVar. Correlatively, *CBL* c.2345C>T failed to segregate with the disease in a large dominant family (Figure S1).

### Molecular Dynamic (MD) Simulations for Novel Variants

**Figure 2:**
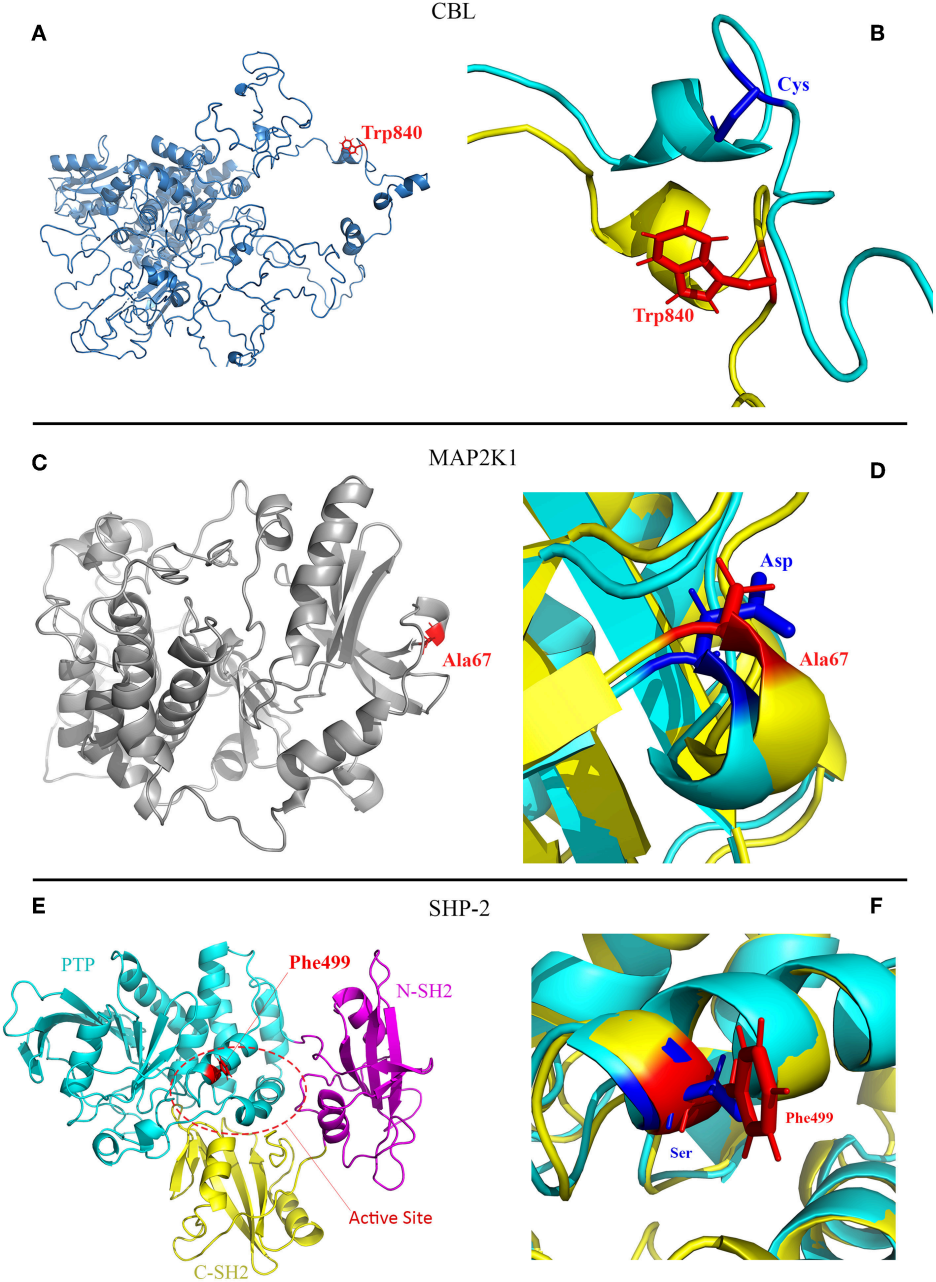
Molecular modeling scheme of the CBL p.Cys840Trp and MAP2K1 p.Asp67Ala mutants, and crystal structure of the SHP-2 p.Ser499Phe (PDB:2SHP). **(A)** CBL protein with mutated residue (Trp840) colored red. The non-conservative substitution of Cys840 negatively charged and hydrophilic with Trp840 non-polar and hydrophobic may impact binding interaction, stability and the flexibility of the protein; **(B)** Zoom of the CBL mutation site comparing the configuration of the wild type and mutant protein and illustrating the flexibility of the structures. **(C)** MAP2K1 protein showing the mutated residue (Ala67) colored red. The substitution of Asp67 negatively charged and hydrophilic with Ala67 non-polar and hydrophobic potentially impact binding interactions; **(D)** Zoom of the MAP2K1 mutation site comparing the configuration of the wild type and mutant structures. **(E)** Crystal structure of SHP-2 including three domains of the protein: PTP (cyan), C-SH2 (yellow) and N-SH2 (pink). The mutated residue (Phe499) colored red is located in active site (Lee et al., 2005). The substitution of the polar and hydrophilic Ser499 with the non-polar and highly hydrophobic Phe499 in the active site of the protein potentially impact binding interactions and the stability of the new structure; **(F)** Zoom of the SHP-2 mutation site comparing the configuration of the wild type and mutant structures.

Molecular dynamic (MD) simulations (Figure 2) showed that: for CBL, substitution of the negatively charged and hydrophilic amino acid Cys840 with the non-polar and hydrophobic amino acid Trp840 could impact binding interactions, stability and the flexibility of the protein.

For MAP2K1, substitution of the negatively charged and hydrophilic amino acid Asp67 with the non-polar and hydrophobic amino acid Ala67 could impact binding interactions; for SHP-2, substitution of the polar and hydrophilic amino acid Ser499 with the non-polar and highly hydrophobic amino acid Phe499 in the active site of the protein could impact binding interactions and the stability of structure.

### Genotype-Phenotype Correlations

**Figure 3:**
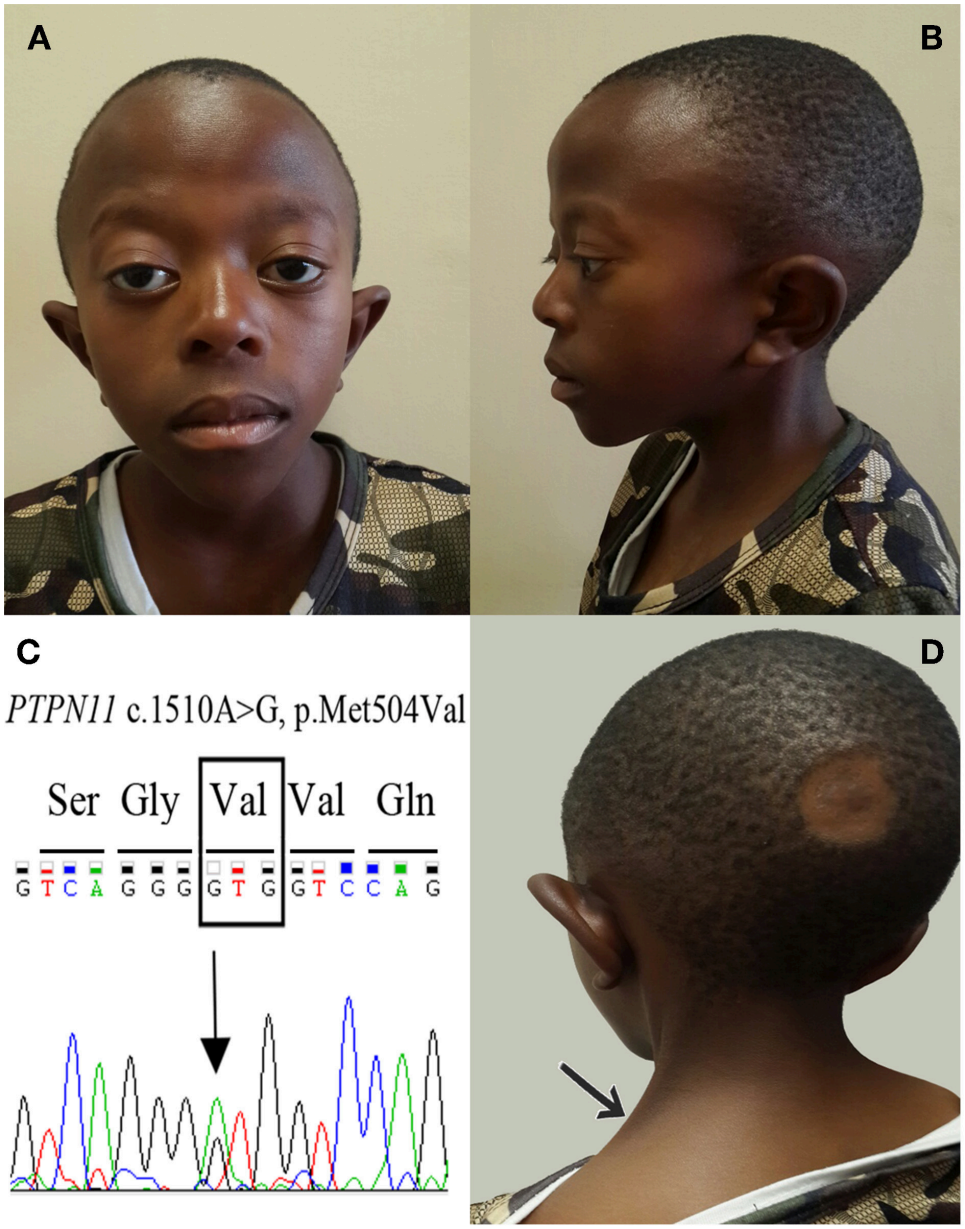
Craniofacial features of an 11-year-old boy with NS and *PTPN11* c.1510A>G (p.Met504Val) variant. **(A)** Frontal views showing a triangular face with pointed chin; tall forehead; bilateral ptosis, predominantly on the right; sparse eyebrows; epicanthic folds and protruding ears. **(B)** Lateral view showing high anterior hairline with low-set and posteriorly rotated ears. **(C)** Electropherogram of the *PTPN11* c.1510A>G (p.Met504Val) variant. **(D)** Posterior view with the arrow indicating webbing of the neck.

Correlations between the five variants that were predicted to be pathogenic, and NS related phenotypes are presented in Table 4. Comparisons showed that patients with variants in *MAP2K1* were diagnosed earlier (mean age at diagnosis: 1 year) and presented with more typical clinical features of NS, followed by patients with variants in *PTPN11* (mean age at diagnosis: 3.3 years; Figure 3). The patient with a variant in *CBL* was found to have more discreet clinical features (Table 5).

**Table 4:** Summary of clinical features in mutation-positive patients.

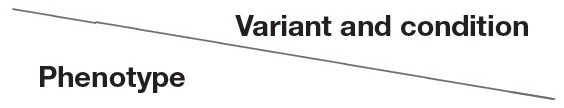	**Patient 1**	**Patient 2**	**Patient 3**	**Patient 4**	**Patient 5**
	***PTPN11* c.1496C>T NS**	***PTPN11*c.1510A>GNS**	***CBL* c.2520T>G NS**	***MAP2K1*c.200A>CNS**	***MAP2K1* c.389A>G CFC**
Gender	M	M	M	M	M
Age at diagnosis	6 years	20 months	17 months	12 months	12 months
Family history	+	+	–	+	–
**Antenatal and birth features**					
Polyhydramnios	–	Unavailable	–	–	+
Prenatal/neonatal lymphatic abnormalities	–	–	–	+	–
Birth weight (centile)	50th	10th	< 3rd	90th	25th
Birth length (centile**)**	50th	Unavailable	< 3rd	90th	Unavailable
**Short stature**	+	+	+	+	+
**Craniofacial dysmorphism**					
Typical facial features	+	+	–	+	+
Mild facial dysmorphism	–	–	+	–	–
**Neck and thoracic features**					
Webbed/short neck	+	+	+	+	+
Pectus deformity of the chest	–	–	–	+	+
**Congenital heart defect and ecg**					
Pulmonary valve stenosis	+	–	–	+	–
Hypertrophic cardiomyopathy	–	+	–	–	–
Coarctation of the aorta	–	–	+	–	–
Left axis deviation on ECG	+	–	–	+	–
Aortic valve stenosis	–	+	–	–	–
Mitral valve incompetence	–	+	–	–	–
Bicuspid aortic valve	–	–	+	–	–
**Neurology, education, and behavioral abnormalities**					
Motor delay	–	+	–	+	+
Speech delay	–	–	–	+	+
Mild ID	–	–	N/A	+	+
Learning difficulties	–	–	N/A	+	+
Hyperactivity	+	–	N/A	–	–
ADHD	–	–	N/A	–	–
Self-injury	–	–	–	–	+
**Eye abnormalities**					
Strabismus	–	–	–	+	–
**Auditory abnormalities**					
Conductive hearing loss	–	–	–	–	–
**Renal abnormalities**					
Undescended testis in males	+	–	–	+	–
Duplex collection system	–	–	–	+	–
**Gastro-intestinal abnormalities**					
Mild feeding difficulties	+	–	–	–	–
Severe feeding difficulties	–	–	–	–	+
Pyloric stenosis	–	–	–	–	–
**Dermatologic abnormalities**					
Cafe-au-lait spots	–	–	–	+	–
Pigmented naevi	–	–	–	–	+
Curly hair	+	–	+	–	+
**Hematology and oncology**					
Bleeding diathesis	+	+	–	–	–
Abnormal PT & PTT	–	–	–	+	–
Desmoid cyst	–	–	–	+	–

*+: Feature present; –: Feature absent; N/A: not applicable*.

**Table 5:** Comparisons of clinical features associated with the three genes identified.

**Characteristics**	***PTPN11* (*n* = 2)**	***CBL* (*n* = 1)**	***MAP2K1* (*n* = 2)**
Mean age at diagnosis (year)	3.3	1.4	1
Positive family history	2 (100%)	0	1 (50%)
Antenatal features	0	0	2 (100%)
Short stature	2 (100%)	100%	2 (100%)
Typical dysmorphic features	2 (100%)	0	2 (100%)
Webbed/short neck	2 (100%)	100%	2 (100%)
Pectus deformity of the chest	0	0	2 (100%)
Congenital heart defects	2 (100%)	100%	2 (100%)
Pulmonary valve stenosis	1 (50%)	0	1 (50%)
Hypertrophic cardiomyopathy	1 (50%)	0	0
Coagulopathy	2 (100%)	0	1 (50%)
Skin features	0	0	2 (100)
Intellectual disability	0	0	2 (100)

## Discussion

This study provides a unique insight into the clinical and molecular profiles of South African individuals affected by NS, a rare attempt to comprehensively describe this condition in Africa. Patients were diagnosed relatively late, which could be explained by at least three factors: Firstly, the diagnosis of NS was hardly hypothesized and explored by clinicians in prenatal settings, partly due to the absence of prenatal molecular diagnostic testing for RASopathies in South Africa. A prenatal detection rate of 17.3% was reported elsewhere (Croonen et al., 2013). Secondly, in South Africa and Africa at large, the scarceness of trained medical geneticists often results in initial misdiagnosis. Finally, age-related variability in NS physical features makes its clinical diagnosis less easy for some professionals: moderate-to-severely affected individuals would be expected to be diagnosed by childhood, and mildly affected individuals in adulthood following either cardiac decompensation or cascade screening after the birth of a severely affected child (Van der Burgt, 2007; Roberts et al., 2013). Indeed, in the present study, all adult patients were diagnosed with NS during their assessment in the cardiology department. The developmental motor milestone in this cohort of South African patients was comparable to that reported in the literature, with 61.5% of patients being able to walk by the age of 18 months (Sharland et al., 1992). Half (50%) of our patients were able to speak simple two-word sentences before the age of 24 months, far below the average age (31 to 32 months) of simple two-word sentences in NS reported by other authors (Pierpont, 2016). Nevertheless, these differences may be considered with caution due to the small size of our cohort and the fact that only screening measures were used for the evaluation of milestones in the present study. The dysmorphology in NS also varies with ethnicity: a collaborative effort investigating NS-associated physical features in 125 individuals from diverse populations found that, the three most common physical features, present in >70% of individuals were: widely spaced eyes (≥80%), low-set ears (>80%) and short stature (>70%); ptosis was less common in black Africans (63%), and webbed neck less common in Asians (Kruszka et al., 2017). In the present study, Black Africans were found to have the most distinctive features, with epicanthic folds, previously reported to be very common in the general black South African population (Christianson et al., 1995), being the most common feature. However, caution should be observed when interpreting the high frequency of epicanthic folds in this study, as it may be more suggestive of a common variant in the general population than a distinctive feature of NS in South African patients. Congenital heart defects represent a major cause of morbidity and mortality in affected individuals with NS of all age groups (Prendiville et al., 2014). As equally found in this study, CHD are reported in 50–80% of individuals affected with NS, the most common CHD being PS ((Hickey et al., 2011); (Prendiville et al., 2014)).

The molecular detection rate in this study was relatively low (31.2%), compared to the expected >70% when using whole exome sequencing (WES) or a comprehensive multigene panel testing (Aoki et al., 2016). It is unlikely that our lower detection rate could be attributed to inappropriate phenotyping, in view of the stringent patient selection process applied. This suggests that genotyping these patients using other sequencing methods such as WES may allow identification of pathogenic variants in genes not investigated in this study, particularly in the large family (Figure S1) with strong clinical features of NS and no mutation identified. All the variants detected were missense, in accordance with available data on NS (Tartaglia et al., 2002; Nava et al., 2007; Martinelli et al., 2010). Interestingly, we identified a pathogenic variant (*MAP2K1* c.389A>G; p.Tyr130Cys) known to be frequently associated with Cardiofaciocutaneous syndrome (Rodriguez-Viciana et al., 2006). This patient was initially labeled with the diagnosis of NS at the age of 12 months, but the progression of clinical features later favored revision of the diagnosis to Costello syndrome. This case illustrates the challenges in clinical diagnosis of some NS patients, due to overlapping features with other RASopathies. Variability in phenotypic expression, high genetic heterogeneity and low mutation frequency in several NS genes are among the difficulties in establishing consistent correlations between known causative genes or variants and specific phenotypes. Our findings are consistent with the literature, with a positive association between short stature, PS, coagulopathy, pectus deformities of the chest, and variants in *PTPN11* (Tartaglia et al., 2002; Yoshida et al., 2004). Similar to previous reports, patients with variants in *MAP2K1* in our study had typical craniofacial features and skin manifestations of NS (Nava et al., 2007; Nyström et al., 2008). To date, very little is known about genotype-phenotype correlations in NS patients with variants in *CBL*. In addition to presenting with less typical craniofacial features, our patient with a *CBL* variant had a cardiovascular phenotype characterized by a combination of bicuspid aortic valve and coarctation of the aorta, which are infrequently associated with NS.

## Conclusion

This first application of targeted NGS for the molecular diagnosis of NS in South Africans suggests that clinical characteristics and genotype-phenotype correlations found in affected individuals are generally similar to those reported in other populations. Therefore, careful phenotyping based on existing diagnostic criteria can effectively enable the diagnosis of most NS-affected individuals in South Africa. The use of targeted NGS in the present study have allowed for detection of novel variants in genes infrequently associated with NS in other populations. Further studies of a larger African cohort with NS, ideally using WES, are needed.

## Ethics Statement

The study was performed in accordance with the Declaration of Helsinki and with the approval of the Faculty of Health Sciences Human Research Ethics Committee, University of Cape Town (HREC: 449/2016). Written informed consent was obtained from the parents and/or the patient prior to their involvement into the study, including permission to publish photographs.

## Author Contributions

CT-N, AE, and AW contributed to conception and design of the study. CT-N and AW collected data; CT-N, GA, and CB performed molecular analysis and interpretation of data. CT-N wrote the first draft of the manuscript and CB wrote a section of the manuscript. All authors contributed to manuscript revision, read and approved the submitted version.

### Conflict of Interest Statement

The authors declare that the research was conducted in the absence of any commercial or financial relationships that could be construed as a potential conflict of interest.

## Supplementary Material

Figure S1 Pedigree of a family with a dominant inheritance pattern of NS, but no pathogenic variant found in the 14 genes investigated. Affected individuals (black) presented with typical craniofacial features, short stature, pectus deformities of the chest, webbed or short neck, congenital heart defect and café au lait spots. The variant CBL c.2345C>T (p.Pro782Leu) identified in the proband (IV-2), classified as uncertain significance in ClinVar, did not segregate with the disease in the family: five first or second-degree relatives were screened for the detected variant in addition to the index case, including three clinically affected and two non-affected individuals. The number beneath deceased family members indicates their age at death.

Table S1 Summary of clinical features identified in the cohort of 26 patients.
