# Supplementary figures and images for: Noonan Syndrome in South Africa: Clinical and Molecular Profiles
Source: Front Genet. 2019 Apr 16;10:333. doi: 10.3389/fgene.2019.00333 (PMC6477999; doi:10.3389/fgene.2019.00333)

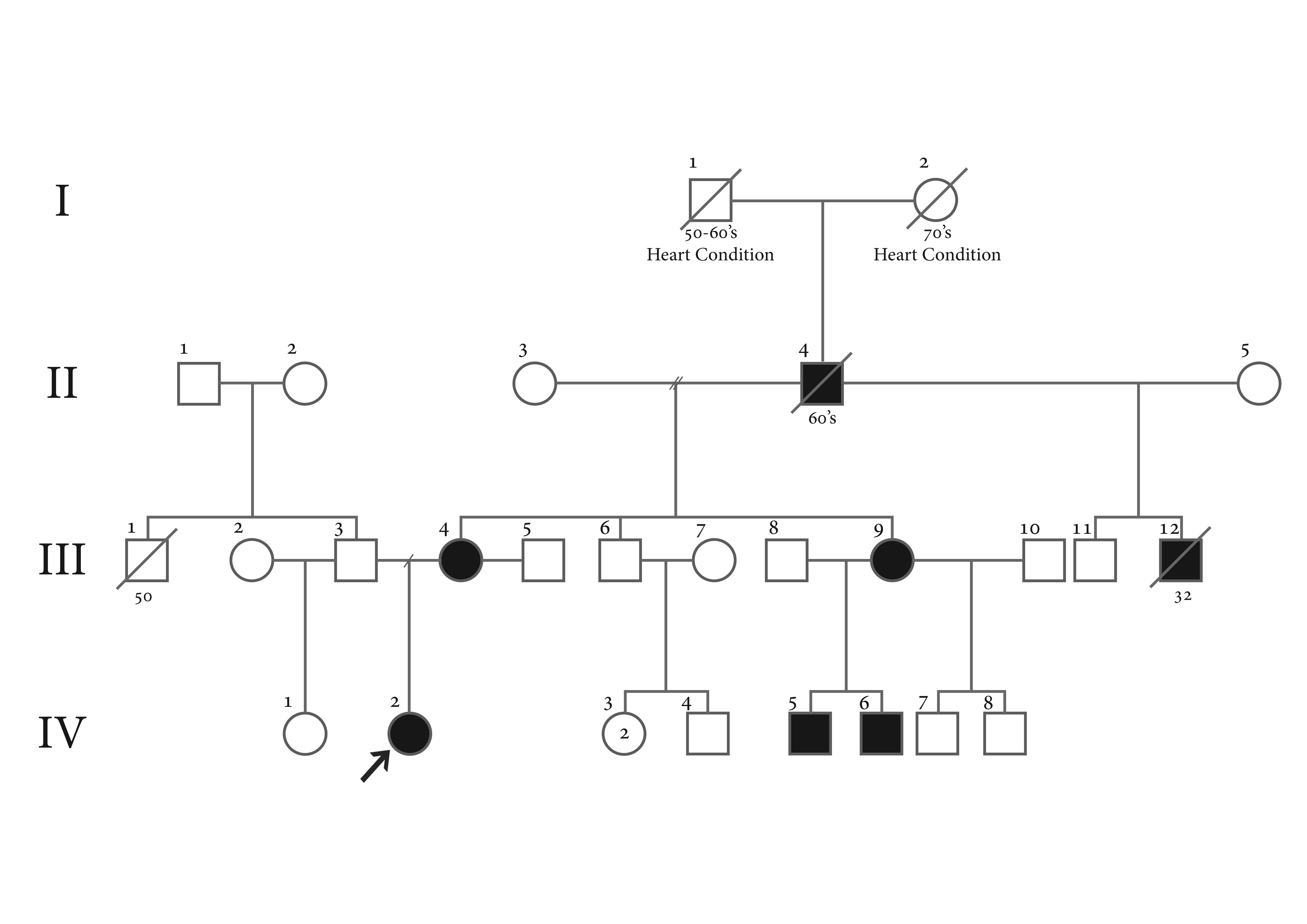

Supplement: Figure S1 — Pedigree of a family with a dominant inheritance pattern of NS, but no pathogenic variant found in the 14 genes investigated. Affected individuals (black) presented with typical craniofacial features, short stature, pectus deformities of the chest, webbed or short neck, congenital heart defect and café au lait spots. The variant CBL c.2345C>T (p.Pro782Leu) identified in the proband (IV-2), classified as uncertain significance in ClinVar, did not segregate with the disease in the family: five first or second-degree relatives were screened for the detected variant in addition to the index case, including three clinically affected and two non-affected individuals. The number beneath deceased family members indicates their age at death. [file Image_1.TIF]
