# Supplementary material for: Noonan Syndrome in South Africa: Clinical and Molecular Profiles
Source: Front Genet. 2019 Apr 16;10:333. doi: 10.3389/fgene.2019.00333 (PMC6477999; doi:10.3389/fgene.2019.00333)
Supplement: Table S1 — Summary of clinical features identified in the cohort of 26 patients. [file Table_1.docx]

**Table S1. Summary of clinical features identified in the cohort of 26 patients**

| **Features identified** | | | | | **Frequency**  **(n = 26)** | **Proportion (%)** |
| --- | --- | --- | --- | --- | --- | --- |
| **Short stature** | | | | | 21 | 80.8 |
| **Central Nervous System** | Agenesis of the Corpus Callosum | | | | 1 | 3.8 |
| **Eye** | Strabismus | | | | 4 | 15.4 |
|  | Myopia | | | | 3 | 11.5 |
|  | Nystagmus | | | | 2 | 7.7 |
|  | Astigmatism | | | | 1 | 3.8 |
| **Auditory** | Conductive hearing loss | | | | 3 | 11.5 |
|  | Sensorineural hearing loss | | | | 1 | 3.8 |
| **Oral & Dental** | High arched palate | | | | 11 | 42.3 |
|  | Dental malocclusion | | | | 11 | 42.3 |
|  | Dental caries | | | | 2 | 7.7 |
|  | Widely spaced teeth | | | | 1 | 3.8 |
|  | Overcrowded teeth | | | | 1 | 3.8 |
| **Chest** | pectus excavatum (PE) | | | | 6 | 23.1 |
|  | Pectus carinatum (PC) | | | | 4 | 15.4 |
|  | PC superiorly & PE inferiorly | | | | 1 | 15.4 |
| **Musculoskeletal (other than chest)** | Spine | | Scoliosis | | 5 | 19.2 |
|  |  |  | Lumbar hyperlordosis | | 1 | 3.8 |
|  | Limbs | | Cubitus valgus | | 2 | 7.7 |
|  |  |  | Talipes equinovarus | | 1 | 3.8 |
|  |  |  | High arched feet sole | | 1 | 3.8 |
|  | Joints | | Hyperlaxity | | 3 | 11.5 |
|  |  |  | Contracture | | 2 | 7.7 |
|  | Other | | 5^th^ finger clinodactyly | | 1 | 3.8 |
|  |  |  | Overlapping 2-3 toes | | 1 | 3.8 |
|  |  |  | Fused ribs | | 1 | 3.8 |
| **Gastrointestinal** | Feeding  difficulties | | Poor suck & prolonged  feeding time | | 3 | 11.5 |
|  |  |  | Severe feeding issues with tube-feeding for > 3 weeks | | 3 | 11.5 |
|  |  |  | Very poor suck & slow feeding with recurrent vomiting | | 2 | 7.7 |
|  | Pyloric stenosis | | | | 1 | 3.8 |
| **Genitourinary** | Undescended testis | | | | 7/15 males | 46.7 |
|  | Duplex left collecting system | | | | 1 | 3.8 |
|  | Multicystic kidney | | | | 1 | 3.8 |
| **Dermatology** | Thick curly hair | | | | 15 | 57.7 |
|  | Skin | Cafe-au-lait spots | | | 9 | 34.6 |
|  |  | Pigmented naevi | | | 3 | 11.5 |
|  |  | Keratosis pilaris atrophicans faciei | | | 1 | 3.8 |
|  |  | Axillary freckling | | | 1 | 3.8 |
|  |  | Hypopigmented facial lesion | | | 1 | 3.8 |
| **Haematology & Oncology** | Easy bruising | | | | 14 | 53.8 |
|  | Abnormal PT & PTT | | | | 1 | 3.8 |
|  | Mild thrombocytopenia | | | | 1 | 3.8 |
|  | Desmoid cyst | | | | 1 | 3.8 |
| **Lymphatic** | Lymphedema | | | | 3 | 11.5 |
| **Neurology, Cognition&**  **Behaviour** | Learning difficulties | | | | 11 | 42.3 |
|  | Mild intellectual disability | | | | 7 | 26.9 |
|  | Severe intellectual disability | | | | 1 | 3.8 |
|  | Behavioural issues | | | Hyperactive | 5 | 19.2 |
|  |  |  |  | ADHD | 4 | 15.4 |
|  |  |  |  | Self-injury | 1 | 3.8 |
